# Mental health status in healthcare workers during COVID-19 pandemic: An online questionnaire study in the southwest Iran

**DOI:** 10.1371/journal.pone.0298058

**Published:** 2024-04-26

**Authors:** Sara Sarvandian, Shima Hosseinpour, Khojasteh Hoseinynejad, Reza Davasaz Irani, Sirus Pakseresht, Zahra Rahimi

**Affiliations:** 1 Department of Biostatistics and Epidemiology, Clinical Sciences Research Institute, School of Health, Ahvaz Jundisapur University of Medical Sciences, Ahvaz, Iran; 2 Expert in Charge of Preventing Substance and Alcohol Use, Vice Chancellor for Health, Khuzestan Health Center, Ahvaz Jundishapur University of Medical Sciences, Ahvaz, Iran; 3 Faculty of Medicine, Department of Physiology, Persian Gulf Physiology Research Center, Medical Basic Sciences Research Institute, Ahvaz Jundishapur University of Medical Sciences, Ahvaz, Iran; 4 Department of Mental Health, The Health Center of Khuzestan Province, Ahvaz Jundishapur University of Medical Sciences, Ahvaz, Iran; 5 Department of Psychiatry, Jundishapur University of Medical Sciences, Ahvaz, Iran; 6 Department of Biostatistics and Epidemiology, Clinical Sciences Research Institute, School of Public Health, Hearing Research Center, Ahvaz Jundishapur University of Medical Sciences, Ahvaz, Iran; Public Library of Science, UNITED KINGDOM

## Abstract

**Objectives:**

This study aimed to evaluate Mental Health Status, such as stress, anxiety, or depression symptoms, during the Covid-19 pandemic in healthcare workers at Ahvaz Jundishapur University of Medical Sciences.

**Methods:**

This study is an online cross-sectional study conducted on healthcare professionals at Ahvaz Jundishapur University of Medical Science from November 25, 2020, to March 30 2021. PHQ9 and Kessler collected outcome variables of depression, anxiety, and psychological distress questionnaires.

**Results:**

In total, 2552 healthcare workers in 24 hospitals and 212 Comprehensive health centers were enrolled in this study. The mean age of participants was 37.94 ± 8.07 years, and 25.3% were male. There was a significant difference between the mean Kessler and PHQ9 Scale scores on males and females (P< 0.001). Also, the results showed a significant difference between depression, anxiety, and stress and working in the intensive care unit. According to the result of the Kessler scale, 27% of participants had moderate to severe. Most respondents (65.5%) in all professions had moderate to severe mental distress scores according to the Kessler scale. The logistic regression model results illustrated the relationship between depression and anxiety with gender, workplace, support from families, and collogue job satisfaction, and feeling the stress of media coverage of COVID-19 were statistically significant (P< 0.05).

**Conclusions:**

The present study revealed that medical and health workers suffer from mental health problems. In this study, up to one-fifth of medical staff had stress, anxiety, or depression symptoms, and more than half had psychological distress. Low educational level, lack of family support, lack of colleague’s support, and being a female were the significant risk factors for stress, anxiety, and psychological distress in medical staff.

## Introduction

In December 2019, the COVID-19 pandemic was reported in Wuhan, China. The novel coronavirus spread worldwide and caused COVID-19 disease [1,2]. It is a new type of the coronavirus family and has spread worldwide [3]. On March 11, 2020, World Health Organization (WHO) declared the disease a "Global Pandemic" [4,5]. The virus spreads through respiratory droplets, bodily fluids, fecal-oral, direct contact, and environmental surfaces [6,7].

COVID-19 is still a considerable threat to healthcare workers at the front lines of care, and they are thus more susceptible to infection [8]. According to a study in China, many healthcare workers were confirmed to have COVID-19 infections after working in COVID-19 centers [9].

Mental health is the overall wellness of thinking, regulating feelings, and behaving. Sometimes people experience a significant disturbance in this mental functioning. A mental disorder may be present when patterns or changes in thinking, feeling, or behaviour cause distress or disrupt a person’s ability to function [10]. During the pandemic, healthcare workers were under intense pressure resulting from long working hours, infection risk, a lack of contact with their families, fatigue, burnout, and isolation [11–13]. This situation can lead to anxiety, depression, stress disorder, and mental health problems [14–16]. Mental health problems of Healthcare workers can cause them to lose their attention, cognitive function, and clinical decision-making [17,18].

On the other hand, studies have shown that acute stress in crises can affect overall health [19,20]. Hence, mental health problems in Healthcare workers during the COVID-19 epidemic have become an urgent public health concern [21]. Numerous review studies have examined the effect of COVID-19 on the mental health of Healthcare workers and showed that mental disorders are highly prevalent among these groups during the pandemic [22–25]. The Huang et al. study showed that the rate of anxiety in medical staff was 23%. In this study, anxiety was higher in women than men and more in nurses than doctors [26]. Repon et al. examined the mental health of healthcare workers. They demonstrated that COVID-19 affected their psychological health, and mental health problems are significantly associated with gender, level of education, smoking, job, area of residence, economic status, and workplace [27].

In Iran, the first cases of confirmed COVID-19 were diagnosed using the Polymerase Chain Reaction (PCR) test on February 19, 2020, in Gom [28]. From the beginning of the COVID-19 pandemic to May 2022, 7 million people were infected approximately, and 141,271 death occurred in Iran [29].

In the present study, due to the high prevalence of COVID-19 and to minimize face-to-face interactions, we preferred to use the online method instead of paper questionnaires. Also, the advantages of online questionnaires include more convenience, more honest answers, accessibility on any device, and cost reduction [30].

Despite the increasing incidence and mortality of this disease in Iran and its psychological effects, especially in the health sector, there is still insufficient scientific evidence on the psychological effects of the COVID-19 pandemic on health staff in Iran. Therefore, this study aimed to evaluate mental health status and related factors during the Covid-19 pandemic in healthcare workers at Ahvaz Jundishapur University of Medical Sciences(AJUMS). The results of this study can be used in the design of educational, health, and therapeutic interventions.

## Material and methods

### Design study and participant characteristics

A cross-sectional study was conducted for four months, from November 25, 2020, to March 30 2021. The study population was all healthcare workers (clinical and non-clinical staff) working in the 24 hospitals and 212 health centers of Ahvaz Jundishapur University of medical sciences during the COVID-19 pandemic. We surveyed using an online questionnaire administered via a Web-based survey platform.

The participants were informed about the aim of this study. They were given the option that they may or may not join the study. The unwilling participants were excluded from the study. The questionnaire was distributed across Social media. They were invited to complete it, and on the first page of the questionnaire, the participant must agree to informed consent. This study was approved by the Ethics Research Committee of Ahvaz Jundishapur University of Medical Sciences with a code of ethics IR.AJUMS.REC.1399.084. This study was conducted according to the Helsinki Declaration. We surveyed using an online questionnaire administered via a Web-based survey platform.

The questionnaire consisted of basic demographic information, the Persian version of the Patient Health Questionnaire (PHQ-9), Kessler Psychological Distress Scale (Kessler). The questionnaires we filled out and received were included in the analysis.

### Sample size and sampling method

To determine the sample size, the proportion estimating formula was used, in which, referring to a previous study in Iran [31], the prevalence of anxiety was considered to be 23.6%. α = 0.05 and d = 0.025 were considered, and the final sample size was 3349. Due to the use of the cluster method in sampling, the design effect was considered 1.75, and the attrition was also considered 35%. The post hoc power calculated for work in the emergency district was obtained as 89%.

Sampling was done in a two-stage method. There are 65 medical centers and 949 health centers in 23 cities of Khuzestan province. This study randomly selected 24 of 65 medical centers and 212 of 949 health centers. A total of 236 centers with about 7,000 personnel were selected. In the second stage, 3,500 people were selected from the list of personnel using the regular random method within each center. Then, from the list of 7,000 personnel from selected medical and health centers, the online questionnaire was sent to 3500 people randomly. 2552 personnel completed the questionnaire (72.9 response rate).

### Instruments

#### General questionnaire

The demographic questionnaire items include age, gender, level of education, and job information, including Job type, workplace, job satisfaction, years of work experience, and work shift. The general questionnaire also included other information consisting of family and friends support, boss and colleagues, and feeling the stress of media coverage of Corona.

#### PHQ9 questionnaire

This questionnaire was a self-reported scale of the PHQ with nine items applied to determine the severity of depressive symptoms such as insomnia and depressed mood [32,33]. Each of the nine items is divided into a four-point Likert scale ranging from: “not at all,” “several days,” “more than half the days,” and “nearly every day.” PHQ-9 comprises five categories ranging from 0–4 (without depressive symptoms), 5–9 (mild depressive symptoms), 10–14 (moderate depressive symptoms), 15–19 (moderately-severe depressive symptoms), and 20–27 (severe depressive symptoms). The total score ranged from 0 to 27 [34,35]. To use the logistic regression model, we divided people into two classes without mental disorders and with a mental disorder (of any severity). The internal consistency of the Persian version of PHQ9 by Cronbach’s alpha coefficient was calculated, and correlation coefficients of 0.89 were confirmed [36]. In our study, ICC was calculated due to the use of the online version of the questionnaire, which was 0.86 in this questionnaire.

#### Kessler questionnaire

Ten-item Kessler Psychological Distress Scale with items asking participants about the signs and symptoms of distress. Each item in the five-point Likert Scale has “none of the time,” “a little of the time,” “some of the time,” “most of the time,” or “all of the time” total minimum and maximum scores for the Kessler distress scale are 10 and 50, respectively [37]. This Scale is psychological distress based on a framework that includes behavioural, emotional, cognitive, and psychophysiological manifestations [38]. Kessler Psychological Distress Scale comprises four categories include 10–19 (no psychological distress), 20-24(mild psychological distress), 25–29 (moderate psychological distress), and 30–50 (severe psychological distress) [39]. To use the logistic regression model, we divided people into two classes without mental disorders and with a mental disorder (of any severity). Cronbach’s alpha reliability coefficient of the Persian Scale was 0.93 [40]. In our study, the ICC obtained in the online version of the questionnaire was 0.91.

This study measured depression, anxiety, and stress levels using the PHQ9 and the Kessler Scale.

### Statistical analysis

The data were analyzed by using SPSS 23 software. We used mean± standard deviation, number, and percent to describe the data. A t-test is a statistical test used to compare the means of Kessler and PHQ9 scores in two groups (Gender, work in the emergency district, Shift work, Job satisfaction, pressure due to mass media, Supported by Family and friends, Supported by colleagues and boss). An ANOVA test was used to determine the difference in the mean between two or more groups. We applied One-way ANOVA analysis, education level, place of work, and type of job as the independent and mean score of Kessler and PHQ9 as dependent variables. Before conducting the ANOVA test, the conditions of parametric tests were examined. The normality of the distribution of the variables was checked using the Kolmogorov–Smirnov test, and then Levene’s test was used to check the homogeneity of variances. We also used Cohen’s F statistic to calculate the effect size. Tukey’s post hoc test was used for pairwise comparison.

The Chi-square test was used for categorical outcomes. There are two major assumptions of the Pearson chi-square test. The first one is individual observations should be independent of each other. The second important chi-square assumption is that the expected cell frequencies should not be too small. The Pearson correlation coefficients determined relationships between questionnaire scores and continuous variables. Statistical significance was considered less than 0.05. We used logistic regression analysis to investigate the effect of risk factors. We first conducted univariate logistic regression analysis. We then fitted a multivariate logistic regression, including those independent variables with a resulting odds ratio (OR) with a p-value less than 0.2 from the univariate models. Logistic regression has five assumptions: The logistic regression assumes minimal or no multicollinearity among the independent variables. The Logistic regression assumes that the independent variables are linearly related to the log of odds. Logistic regression usually requires a large sample size to predict correctly. Logistic regression, which has two classes, assumes that the dependent variable is binary and ordered logistic regression requires the dependent variable to be ordered. The Logistic regression assumes the observations to be independent of each other. The study aims to investigate the relationship between the independent variables, such as demographic factors, job factors, and psychological support from colleagues and family with anxiety, depression, and mental health.

The Ethics Research Committee of Ahvaz Jundishapur University of Medical Sciences approved this study.

## Results

### Participant’s characteristics

There were 2552 participants in our study. The mean age of participants was 37.94 ± 8.07 years. According to the results, participants’ mean years of working experience was 12.66±8.16, and the mean of monthly working hours was 169.97±179.9. The Characteristics of the participants are shown Table 1.

**Table 1:** Demographic characteristics of medical and healthcare workers (N = 2552).

Variables		n	%
**Gender**	Male	646	25.3
	Female	1906	74.7
**Education**	High school or below	58	2.3
	Diploma	345	13.5
	Bachelor	1661	65.1
	Master	360	14.1
	Ph.D./ MD	87	3.4
	Specialist	41	1.6
**Workplace**	Hospital	1005	39.4
	Medical and health care center	365	14.3
	Health center	1182	46.3
**Profession**	Doctor	111	4
	Nurses	673	26.4
	Medical caregiver	139	5.5
	Technician	221	8.7
	Midwife	360	14.1
	Health administrators	385	15.1
	Social worker and psychologist	112	4.4
	Others	556	21.8
**Work in emergency district**	No	2072	81.2
	Yes	480	18.8
**Shift work**	No	1419	55.6
	Yes	1133	44.4
**Supported by families and friends**	No	429	16.8
	Yes	2123	83.2
**Supported by boss and colleagues**	No	1600	62.7
	Yes	952	37.3
**Job satisfaction**	No	926	36.3
	Yes	1626	63.7
**Feel the stress of media coverage of Corona**	No	582	22.8
	Yes	1970	77.2

### Prevalence of mental health

According to the results obtained from the PHQ9 questionnaire, 7.8% had severe depression, 6.5% had moderate to severe depression, 13.2% had moderate depression, and 27.5% had mild depression. Also, 45% of the participants had no symptoms of depression. The results obtained from Kessler’s questionnaire showed that 22% were in severe mental distress, 43.5% were moderate, and 33.7% were in the mild group; the rest were in the healthy group.

### Risk factors influencing mental health

Pearson’s correlation analysis was used to identify the correlations between the results from the responses of the Healthcare workers. There was no significant correlation between the Kessler scores and age, work experience, and Working hours per month (p>0.05). Similarly, there was no significant correlation between the PHQ9 scores and age, work experience, and working hours per month (P> 0.05). The results in the gender group showed that there is a significant positive correlation between age and Kessler’s score in women (P> 0.05). Still, the same group had a negative correlation between age and PHQ9 score (P> 0.05). Also, a negative correlation was shown in the male group between the PHQ9 score and work experience (P> 0.05).

According to Table 2, the independent t-test showed a significant difference between a score of PHQ9 and Kessler scales and gender, working place, job satisfaction, supported by family and colleagues, and pressure due to mass media. Regarding the result, staff who work shift work or are unsatisfied with their job had depression, anxiety and stress sign, and psychological distress more than others. In addition, pressure due to mass media news, psychological disorders, and Depressive, anxiety, and stress are higher among medical staff. Also, being supported by families, friends, and colleagues can help decrease psychological disorders and depression, anxiety and stress, and Psychological Distress.

**Table 2:** Comparison of depression and anxiety scales between variables categories.

Characteristics	Categories	n	PHQ9			n	Kessler		
			Mean	SD	P value*		Mean	SD	P value*
**Gender**	**Male**	646	5.09	5.61	<0.001	646	21.39	6.19	<0.001
	**Female**	1906	7.79	6.65		1906	18.91	5.7	
**work in the emergency district**	**Yes**	480	8.24	6.84	<0.001	480	18.07	6.3	<0.001
	**No**	2072	7.15	6.33		2072	19.88	5.78	
**Shift work**	**Yes**	1133	8.06	6.64	<0.001	1133	18.15	5.99	<0.001
	**No**	1419	6.34	6.30		1419	20.65	5.63	
**Job satisfaction**	**Yes**	1626	5.26	5.29	<0.001	1626	16.24	5.32	<0.001
	**No**	926	10.33	7.17		926	21.42	5.40	
**pressure due to mass media**	**Yes**	1970	7.88	6.6	<0.001	1970	22.71	5.68	<0.001
	**No**	582	4.47	5.43		582	18.60	5.67	
**Supported by Family and friends**	**Yes**	2123	6.61	6.33	<0.001	2123	20.05	5.97	<0.001
	**No**	429	9.54	6.87		429	17.02	4.96	
Supported by colleagues and boss	**Yes**	952	4.67	5.21	<0.001	952	22.24	5.33	<0.001
	**No**	1600	8.55	6.78		1600	17.94	5.67	

*P-value in t-test.

One-way ANOVA showed a significant difference in the level of education, Place of work, Type of job, and the mean score of Kessler and PHQ9 questionnaire (Table 3). Tukey’s post-hoc test showed that the mean score of the PHQ9 questionnaire in individuals with an education level below a diploma is significantly higher than other education levels. However, in Kessler’s questionnaire, the mean score at the diploma level was higher than the rest of the groups. Also, the mean score of participants with a diploma level showed a significant difference between the Bachelor of Science and doctorate education levels. The mean score of those working in the hospital is significantly higher than the other places in both questionnaires, Kessler and PHQ9. In the work type variable, the severity of depression in Medical caregivers, Social workers and psychologists was significantly lower than in other groups While; based on the results of Kessler’s questionnaire; these groups had more symptoms of distress.

**Table 3:** Comparison of questionnaire scores means between demographical characteristics.

Characteristics	Categories	n	PHQ9					n	Kessler				
			Mean ±SD	95% Confidence Interval		P- value*	Effect size		Mean ± SD	95% Confidence Interval		P–value*	Effect size
				Lower bound	Upper bound					Lower bound	Upper bound		
**Education levels**	under diploma	58	12.26±16.7	7.81	16.71	0.001	0.131	58	16.66±8	12.22	21.10	0.001	0.208
	diploma	345	4.47±5.5	3.37	5.56			345	22.18±5.8	20.93	23.43		
	BS	1661	7.67±8.3	7.02	8.31			1661	18.92±5.9	18.35	19.50		
	Master	360	6.09±7.3	4.89	7.30			360	20.38±4.7	19.38	21.38		
	Doctorate	87	7.45±10.2	4.61	10.29			87	19.90±5.1	17.60	22.21		
	Specialist	41	7.10±11	3.18	11.01			41	18.30±3.9	15.45	21.14		
**Place of work**	Hospital	1005	8.18±6.6	7.36	9	0.003	0.138	1005	17.79±5.8	17.07	18.52	0.001	0.252
	Medical and health care center	365	6.32±5.8	5.10	7.54			365	20.78±5.3	19.67	21.88		
	Health center	1182	6.43±6.4	5.69	7.17			1182	20.65±5.7	19.99	21.30		
**Type of job**	Doctor	111	8.46±6.8	4.65	12.27	0.0290	0.271	111	19.53±4.56	17	22.06	0.001	0.336
	Nurses	673	8.28±6.8	7.25	9.32			673	17.64±5.6	16.78	18.5		
	Medical caregiver	139	2.33±1.5	-1.46	6.12			139	24.33±3.2	16.34	32.31		
	Technician	221	7.81±6.7	4.84	10.79			221	18.18±5.4	15.75	20.61		
	Midwife	360	7.21±6	5.95	8.48			360	19.7±5.5	18.53	20.86		
	Health administrators	385	7.86±8.3	4.16	11.56			385	19.36±6.3	16.54	22.18		
	Social worker and psychologist	112	2.73±3.5	1.02	4.45			112	22.73±4.8	20.37	25.09		
	Others	556	6.68±6	5.6	7.76			556	20.61±5.6	19.61	21.61		

* P-value in ANOVA test.

The relationship between the severity of mental disorders based on Kessler and PHQ9 questionnaires and the variables gender, qualification, workplace, Profession, Work in an emergency district, working shift, supported by families and friends, supported by boss and colleagues, Job satisfaction, Feel the stress of media coverage of COVID 19 using the chi-square test was shown in Table 4.

**Table 4:** The Kessler scale and PHQ9 Scale grades among the healthcare workers’ baseline characteristics.

		PHQ9							Kessler				
Group		Without depression (%)	Mild (%)	Moderate (%)	Moderately- Severe (%)	Severe (%)	χ^2^	P-value*	Mild (%)	Moderate (%)	Severe (%)	χ^2^	P-value*
Gender	Male	388(33.77)	139(19.80)	63(18.75)	28(16.77)	28(14.14)	81.912	0.000^*^	334(38.39)	227(20.32)	85(15.04)	124.932	0.000^*^
	Female	761(66.23)	563(80.20)	273(81.25)	139(83.23)	170(85.86)			536(61.61)	890(79.68)	480(84.96)		
Education	High school or below	12(1.04)	12(1.71)	7(2.08)	12(7.19)	15(7.61)	165.60	0.000^*^	14(1.61)	22(1.97)	22(3.89)	152.493	0.000^*^
	Diploma	230(20)	67(9.56)	28(8.31)	12(7.19)	8(4.06)			195(22.47)	126(11.27)	24(4.24)		
	Bachelor	678(58.96)	476(67.90)	238(70.62)	115(68.86)	154(78.17)			500(57.60)	712(63.69)	449(79.33)		
	Master	178(15.48)	114(16.26)	36(10.68)	16(9.58)	16(8.12)			123(14.17)	190(16.99)	47(8.30)		
	Ph.D./ MD	35(3.04)	24(3.42)	16(4.75)	8(4.79)	4(2.03)			28(3.23)	43(3.85)	16(2.83)		
	Specialist	17(1.48)	8(1.14)	12(3.56)	4(2.40)	0(0)			8(0.92)	25(2.24)	8(1.41)		
Workplace	Hospital	360(31.30)	322(45.93)	158(46.88)	71(42.51)	94(47.72)	84.931	0.000^*^	223(25.72)	463(41.38)	319(56.36)	148.415	0.000^*^
	Medical and health care center	167(14.52)	118(16.83)	36(10.68)	32(19.16)	12(6.09)			140(16.15)	181(16.18)	44(7.77)		
	Health center	623(54.17)	261(37.23)	143(42.43)	64(38.32)	91(46.19)			504(58.13)	475(42.45)	203(35.87)		
Profession	Doctor	40(3.48)	28(3.95)	20(5.93)	12(7.23)	4(2.01)	141.508	0.000^*^	28(3.41)	56(5.01)	21(3.43)	220.006	0.000^*^
	Nurses	258(22.43)	258(36.39)	107(31.75)	55(33.13)	67(33.67)			156(18.98)	372(33.30)	215(35.07)		
	Medical caregiver	214(18.61)	55(7.76)	28(8.31)	28(16.87)	28(14.07)			195(23.72)	100(8.95)	52(8.48)		
	Technician	48(4.17)	35(4.94)	8(2.37)	8(4.82)	8(4.02)			24(2.92)	51(4.57)	32(5.22)		
	Midwife	143(12.43)	115(16.22)	56(16.62)	28(16.87)	20(10.05)			127(15.45)	143(12.80)	92(15.01)		
	Health administrators	134(11.65)	55(7.76)	43(12.76)	15(9.04)	32(16.08)			92(11.19)	120(10.74)	68(11.09)		
	Social worker and psychologist	71(6.17)	23(4.51)	8(2.37)	4(2.41)	4(2.01)			11(1.34)	44(3.94)	57(9.30)		
	Others	242(21.04)	131(18.48)	67(19.88)	16(9.64)	36(18.09)			189(22.99)	231(20.68)	76(12.40)		
Work in emergency district	Yes	202(17.58)	119(16.98)	59(17.51)	44(26.51)	56(28.14)	20.839	.000^*^	116(13.39)	200(17.89)	164(28.87)	54.916	0.000^*^
	No	947(82.42)	582(83.02)	278(82.49)	122(73.49)	143(71.86)			750(86.61)	918(82.11)	404(71.13)		
Working shift	Yes	420(36.55)	357(50.85)	154(45.83)	95(56.89)	106(53.54)	58.032	0.000^*^	285(32.95)	510(45.58)	338(59.51)	99.093	0.000^*^
	No	729(63.45)	345(49.15)	182(54.17)	72(43.11)	92(46.46)			580(67.05)	609(54.42)	230(40.49)		
Supported by families and friends	Yes	1025(89.21)	575(81.91)	257(76.49)	115(69.28)	151(75.88)	71.954	0.000^*^	815(94.11)	896(80.07)	412(72.66)	126.567	0.000^*^
	No	124(10.79)	127(18.09)	79(23.51)	51(30.72)	48(24.12)			51(5.89)	223(19.93)	155(27.34)		
Supported by boss and colleagues	Yes	599(52.09)	206(29.39)	83(24.70)	36(21.56)	28(14.14)	212.182	0.000^*^	482(55.47)	405(36.26)	65(11.48)	284.419	0.000^*^
	No	551(47.91)	495(70.61)	253(75.30)	131(78.44)	170(85.86)			387(44.53)	712(63.74)	501(88.52)		
Job satisfaction	Yes	916(79.72)	422(60.03)	178(52.98)	55(33.13)	55(27.78)	325.989	0.000^*^	746(85.94)	703(62.82)	177(31.33)	442.266	0.000^*^
	No	233(20.28)	281(39.97)	158(47.02)	111(66.87)	143(72.22)			122(14.06)	416(37.18)	388(68.67)		
Feel the stress of media coverage of COVID 19	Yes	745(64.84)	618(88.16)	286(84.87)	146(87.95)	175(87.94)	182.742	0.000^*^	527(60.85)	923(82.56)	520(91.55)	216.092	0.000^*^
	No	404(35.16)	83(11.84)	51(15.13)	20(12.05)	24(12.06)			339(39.15)	195(17.44)	48(8.45)		

*P-value in the chi-square test.

Using the Chi-square test, the comparison showed a significant difference in proportion across the levels of severity of depression on the Kessler scale (χ2 = 30.64, P< 0.001) and PHQ9 scale (χ2 = 20.84, P = 0.001).

Table 5 illustrates the result of univariate and multivariate logistic regression. As a result of the PHQ9 questionnaire, depression was 71% higher in females than males (OR = 1.71, CI 95%: 1.12–2.63, P = 0.013). Also, in Kessler, depression in females was 2.11 times more than in males (OR = 2.11, CI 95%: 1.39–2.23, P = 0.001). The odds of working in a hospital compared with working in health centers was 2.04 (CI 95%: 1.18–3.51, P = 0.011) in PHQ9 was 1.78 (CI 95%: 2.56–6.60, P < 0.05) in Kessler. Lack of boss and colleagues support (OR = 2.47, CI 95%: 1.69–3.61, P< 0.05), Job dissatisfaction (OR = 2.87, CI 95%: 1.92–4.30, P< 0.05) and feeling stress of media coverage of COVID 19 (OR = 3.37, CI 95%: 2.17–5.22, P < 0.05) increase the odds of depression in PHQ9 questionnaire. In the Kessler questionnaire, lack of families and friends support (OR = 3.3, CI 95%: 1.67–6.54, P = 0.001), lack of boss and colleagues support (OR = 1.91, CI 95%: 1.28–2.85, P< 0.002), Job dissatisfaction (OR = 4.11, CI 95%: 2.56–6.60, P < 0.05) and feel the stress of media coverage of COVID 19 (OR = 3.26, CI 95%: 2.10–5.06, P < 0.05) increase the odds of depression.

**Table 5:** The crude and adjusted odds ratio of variables by using the logistic regression model.

Variables		n	PHQ9				Kessler			
			Crude ORs		Adjusted ORs		Crude ORs		Adjusted ORs	
			OR (95%CI)	P- value*	OR (95%CI)	P- value**	OR (95%CI)	P- value*	OR (95%CI)	P- value**
**Age**			.99 (.97–1.01)	.35	-	-	.99 (.97–1.01)	.51	-	-
**Gender**	**Male**	646	1		1		1		1	
	**Female**	1906	2.27 (1.58–3.26)	.0001	1.71 (1.12–2.63)	.013	2.72 (1.9–3.94)	.0001	2.11 (1.39–2.23)	.001
**Education**	**High school or below**	58	2.67 (.45–15.96)	.28	4.90 (.688–34.97)	.11	.83 (.11–6.26)	.86	-	-
	**Diploma**	345	.333 (.09–1.27)	.11	.34 (.090–1.75)	.22	.19 (0.04–0.96)	.04	-	-
	**Bachelor**	1661	.967 (.27–3.48)	.96	.72 (.17–2.98)	.65	.58 (.12–2.77)	.50	-	-
	**Master**	360	.681 (.18–2.58)	.57	.53 (.12–2.35)	.40	.48 (.09–2.42)	.38	-	-
	**Ph.D./ MD**	87	.963 (.21–4.42)	.96	1.15 (.21–6.28)	.87	.53 (.09–3.21)	.50	-	-
	**Specialist**	41	1		1		1		-	-
**Workplace**	**Health center**	1005	1		1		1		1	
	**Medical and health care center**	365	1.326 (.83–2.12)	.24	1.88 (1.27–2.80)	.002	2.60 (1.80–3.78)	.0001	2.67 (1.74–4.10)	.0001
	**Hospital**	1182	1.994 (1.41–2.81)	.0001	2.04 (1.18–3.51)	.011	1.18 (.73–1.91)	.49	1.78 (2.56–6.60)	.0001
**Work experience (years)**			111	.95	-	-	1 (.98–1.02)	.61	-	-
**Working hours per month**			673	.75	-	-	1 (.99–1)	.27	-	-
**Work in emergency district**	**No**	139	1		-	-	1		-	-
	**Yes**	221	1.15 (.77–1.72)	.48	-	-	1.78 (1.13–2.80)	.01	-	-
**Working shift**	**No**	360	1		-	-	1		-	-
	**Yes**	385	1.78 (1.31–2.46)	.0001	-	-	2.05 (1.46–2.89)	.0001	-	-
**Supported by families and friends**	**No**	112	2.32 (1.48–3.64)	.0001	-	-	4.56 (2.49–8.35)	.0001	3.30 (1.67–6.54)	.001
	**Yes**	556	1		-	-	1		1	
**Supported by boss and colleagues**	**No**	2072	3.23 (2.32–4.51)		2.47 (1.69–3.61)	.0001	3.22 (2.29–4.54)	.0001	1.91 (1.28–2.85)	.002
	**Yes**	480	1		1		1		1	
**Job satisfaction**	**No**	1419	3.83 (2.69–5.45)	.0001	2.87 (1.92–4.30)	.0001	5.56 (3.63–8.51)	.0001	4.11 (2.56–6.60)	.0001
	**Yes**	1133	1		1		1		1	
**Feel the stress of media coverage of COVID 19**	**No**	429	1		1		1		1	
	**Yes**	2123	3.72 (2.51–5.53)	.0001	3.37(2.17–5.22)	.0001	3.81 (2.59–5.59)	.0001	3.26 (2.10–5.06)	.0001

*P-value in univariate logistic regression.

**P-value ina univariate multiple regression.

## Discussion

The study aims to evaluate mental health status and related factors during the Covid-19 pandemic in Ahvaz Jundishapur University of Medical Sciences (AJUMS) healthcare workers. According to the Kessler scale, most respondents in all professions had moderate to severe mental distress scores. The results illustrated that the relationship between depression and anxiety with gender, workplace, support from families, collogue job satisfaction, and feeling the stress of media coverage of COVID-19 were statistically significant.

During the COVID-19 pandemic, our survey shows a high prevalence of depression, anxiety, and stress in Healthcare workers regarding PHQ9. According to the Kessler scale, the majority of staff had psychological distress. Our results were similar to a study in China [41]. Also, a study in Taiwan showed that nurses who had direct contact with patients during the SARS epidemic had a higher level of depression [42]. The results of a study in Thailand illustrated that the continuation of the Covid-19 pandemic and the creation of mutations in the virus would be accompanied by anxiety disorders and various types of stress such as exposure to the media, exposure to death and loss, restrictions on movement, and economic problems [43].

In this study, women had more depressive, anxiety, and stress signs than men, according to the PHQ9 Scale. This can be justified by the biological characteristics of women and the fact that most women are more sensitive to issues and problems. Studies have reported conflicting results. A study in China reported that anxiety became a severe and frequent problem in females [41]. On the contrary, a study in Nepal showed that gender had no significant effect on any mental health disorder. The Canadian study showed that symptoms of anxiety and depression were more evident in men [44,45].

Our result illustrated that staff who work in the emergency district had mild depression based on PHQ9. Also, the healthcare workers who had shift work and worked in the hospital had a higher mean score on the PHQ9 questionnaire. The psychological distress might be related to difficulties being safe at work, working under pressure, the long-term workload, and the high risk of exposure to patients with COVID-19 [46].

This study showed a significant difference between the level of education with the depressive, anxiety, and stress signs. Healthcare workers with a lower level of education may be more susceptible to mental disorders because they have less understanding of the pandemic. In line with the results of our study, a study in China showed that people with a lower level of education experience more fear of the pandemic than people with a higher education [47].

According to our study, there was a significant difference between stresses in the type of job. In our study, the level of anxiety and depression in clinical staff (doctors and nurses) was higher than in the rest of the groups. These results were similar to a study of staff in Singapore and China. This health worker group is exposed to psychological problems due to high work pressure and long shifts [46,48].

Based on the results, psychological distress was revealed more than in staff unsatisfied with their job. Depression, anxiety, and stress reveal more than in staff unsatisfied with their job. According to Masanotti, tight working conditions, lack of staff, and dissatisfaction with work trigger distress among healthcare workers [49].

According to our study, family support can help decrease psychological distress and depression, anxiety, and stress. A study in Jordan showed that social support, especially from family and friends, effectively reduced depression and anxiety among healthcare personnel during the COVID-19 epidemic [50]. The same results occur regarding the support of colleagues and the boss in the workplace, which the Canadian study also clearly showed [45].

Our study showed that the support of the boss and colleagues reduces anxiety and depression. Some studies also confirmed these results [8].

A pandemic of infectious diseases such as COVID-19 is a global challenge due to its wide and rapid spread, high mortality, and causing suffering among the people. In this situation, the hospitals are overwhelmed, and there are worries about people’s lives. Healthcare workers will face severe work pressures and be more prone to mental health problems than the rest of the community. Given the important role of healthcare workers in such situations, their mental health should be a priority (19, 44, and 46). Timely screening can be done for individuals to reduce and prevent the progression of mental health problems. Family and community support, regular physical activity programs such as exercise, social interactions with colleagues, reduced work shifts, personal protective equipment, and psychological services will significantly impact healthcare workers’ health during the epidemic.

Our limitations of this study include the following: Using the web-based method has reduced responsiveness. Busy people did not have time to complete the questionnaire and could not enter the study. Those who did not participate in the study may differ in mental health from those who participated. Considering that the type of study is cross-sectional, the causal relationships cannot be proven due to reverse causality bias.

## Conclusion

During the COVID-19 pandemic, medical and health workers might suffer significant mental health problems. In this study, up to one-fifth of medical staff had stress, anxiety, or depressive symptoms, and more than half had psychological distress. Female gender, low education level, and lack of supportive family and colleagues were the most critical risk factors in medical staff stress, anxiety, and psychological distress. We suggest that counselling, educational and therapeutic interventions by psychologists and psychiatrists during the pandemic should be given special consideration to healthcare personnel.
